# Social support seeking and self-efficacy-building strategies in enhancing the emotional well-being of informal HIV/AIDS caregivers in Ibadan, Oyo state, Nigeria

**DOI:** 10.1080/17290376.2015.1126794

**Published:** 2016-02-02

**Authors:** Bernedette Okwuchukwu Okeke

**Affiliations:** ^a^ Ph.D clinical Psychology (Guidance and Counselling), Fontanna International School, 6B Adenuga Street, Kongi, New Bodija, Ibadan, Oyo State, Nigeria

**Keywords:** HIV/AIDS, informal caregivers, enhancing emotional well-being, VIH / SIDA, les aidants naturels, l'amélioration de bien-être émotionnel Nombre de mots: 363

## Abstract

This study examined the relative efficacy of social support seeking (SSS) and self-efficacy building (SEB) in the management of emotional well-being of caregivers of people suffering from HIV/AIDS. It was based at the United States President's Emergency Plan for AIDS Relief (PEPFAR) center in the University College Hospital, Ibadan, Oyo state, being the first and the largest teaching hospital in Nigeria. A 3 × 2 factorial design consisting of treatment and a control group was used. The columns have two levels of gender being male and female caregivers. One-hundred and sixty-five (165) caregivers who were taking care of people that are suffering from HIV/AIDS were purposively selected and randomly assigned to the treatment groups and control. The treatment was carried out for a period of eight weeks. Two null hypotheses were tested, both at .05 levels of significance. Data were collected with the use of standardized intruments rating scale; social support scale, general self-efficacy scale and emotional well-being scale. ANCOVA was used to establish significant treatment effects with the pretest as covariate. Even though SSS and SEB were both found to be effective in enhancing the emotional well-being of informal caregivers in this study when compared to the controls, SSS was significantly more effective than SEB in achieving this goal. Since the HIV/AIDS patients cannot be adequately cared for in the hospital settings due to severe shortages of material, personnel and time, serious efforts should be made by the three levels of the health care system viz: the primary, secondary and tertiary health care systems, to encourage the employment of the psychological management of caregivers of people suffering from HIV/AIDS. Also, the psychologists, clinical psychologists and the significant others should be encouraged to employ this psychological management in the care of HIV/AIDS informal caregivers.

## Résumé

Cette étude a examiné l'efficacité relative de la recherche de soutien social et le renforcement de l'autoefficacité dans le gestion des bien-être émotionnel des aidants de personnes atteintes du VIH / SIDA. Il était basé au centre du Plan d'urgence du Président des Etats-Unies pour le SIDA (PEPFAR) dans L’Hôpital de l’University College, Ibadan, l'État d'Oyo, étant le premier et le plus grand hôpital d'enseignement au Nigeria. Une factorielle 3x2 conception consistant en un traitement et un groupe témoin a été utilisé. Les colonnes ont deux niveaux de genre étant soignants masculins et féminins. Cent soixante-cinq (165) soignants qui prenaient des soignés les personnes qui souffrent du VIH / SIDA ont été choisis à dessein et aléatoirement affectés aux groupes et contrôle traitement. Le traitement a été effectué pendant une période de huit semaines. Deux hypothèses nulls ont été testées, tant au 0,05 niveaux de signification. Les données ont été recueillies à l'aide d’ évaluation des Instruments standardizes - des échelles de notations: des échelles de soutien social; L’ échelle générale d'auto-efficacité et l’échelle de bien-être émotionnel. ANCOVA a été utilisé pour établir des effets de traitement significatifs avec le pré-test que covariable. Même si la recherche de soutien social et le renforcement de l'autoefficacité de soi ont tous deux été trouvé efficaces dans améliorer le bien-être émotionnel des aidants naturels dans cette étude par rapport aux contrôles, recherche de soutien social était significativement plus efficace que le renforcement de l'autoefficacité de soi dans la réalisation de cet objectif. Depuis les patients atteints du VIH / SIDA ne peut être prise en charge adéquate dans les milieux hospitaliers en raison de graves pénuries de matériel, de personnel et de temps, de sérieux efforts doivent être faits par les trois niveaux de la système de santé à savoir: l'enseignement primaire, secondaires et tertiaires; d'encourager l'emploi de la prise en charge psychologique des aidants de personnes atteintes du VIH / SIDA. Aussi, les psychologues, des psychologues cliniques et les autres personnes importantes devraient être encouragés à employer cette prise en charge psychologique dans les soins des aidants naturels du VIH / SIDA.

## Introduction/background

The past two decades have seen both the emergence of AIDS as a new life-threatening disease and its conversion from a rapidly fatal illness into a manageable chronic disease. HIV/AIDS has been a great challenge to the country since the first case of AIDS was identified in Nigeria in 1986. The HIV/AIDS is a life-threatening infectious disease, and major advances in highly active antiretroviral therapy (HAART) have converted the disease into a manageable chronic disease, leading to declines in rates of opportunistic infections and deaths. However, even in this period of highly active therapy, AIDS remains dreadful epidemic and an important cause of morbidity and mortality in many young adult populations in Africa.

The dire situation in many homes and communities in Nigeria is a serious challenge to the work of HIV/AIDS caregiving. Being a HIV/AIDS patient can be a highly emotional experience, thus a system of support is crucial for these individuals, to strengthen their coping strategies when dealing with this disease. The support is often provided not only by professionals, but mostly by the individuals who provide assistance to them at home, regarded as the informal caregivers. They are family and friends of people living with HIV/AIDS (PLWHA), who give individuals living with HIV/AIDS emotional, spiritual and physical care in dealing with the effects of HIV/AIDS on their daily lives. The informal caregivers include significant others like parents, children, relatives, spouse/partners and friends who provide in-home care, usually on an unpaid basis. These informal caregivers share the same constraints, needs and challenges that their relatives living with HIV/AIDS do, such as grief, fear of impending death, stress, burnout and social problems such as poverty, lack of food and proper housing, ill health, inability to access health care treatment for relatives and above all, lack of training and information about the disease, care, prolongation and illness progression.

Unfortunately, only a limited number of resources are designed specifically to help the informal caregivers deal with the effects of HIV/AIDS in Nigeria. Recent reforms in health care systems have made individuals with long-term complex health problems such as HIV/AIDS, to be cared for at home by family members (Canam & Acom 1999). To assist AIDS persons cope with this crisis, many nations have encouraged home-based care (HBC) for persons with HIV/AIDS disease. Many of the diseases/related problems of HIV/AIDS are therefore managed at home, where the informal caregiver has the critical role of helping the person with HIV/AIDS deal with the disease and its psychological sequelae.

In Africa generally, there has been a gradual shift in the model of care of PLWHA from hospital to HBC. Patients are often discharged from the hospital after a short admission period or not admitted at all. This is so because even without the impact of AIDS epidemic, the health care systems in many African countries are battling with the burden of diseases such as malaria, bacterial pneumonia, tuberculosis, diarrhea disease, etc. The numbers of doctors are grossly inadequate, while medications are in extreme short supply and diverted from the ministry of health hospitals to private clinics. Existing infrastructures are totally inadequate to provide in-hospital care. The above factors bring great pressure on hospital personnel to discharge AIDS patients quickly with little or no treatment (Canam & Acom 1999).

The HIV/AIDS caregivers are often witness to the decline and death that they too may someday experience (Binson, Woods, Pollack, Paul, Stall, & Catania 2001). The multiplicity of the taboos associated with HIV/AIDS and the fear of contacting it also pose unique challenges for them (Pearlin, Semple & Turner 1988) all of which affect the emotional well-being of those families or informal caregivers.

Emotional well-being is viewed as the capacity to live a full and creative life, and the flexibility to deal with life's inevitable challenges. Positive emotions serve as markers of optimal well-being (Diener, Sandvick & Parot, 1991). Moments in people's lives when they experience positive emotions such as joy, interest, contentment, love, etc. are moments in which they are not plagued by negative emotions such as anxiety, sadness, anger and despair. Self-efficacy is the confidence that a caregiver has in his/her ability to meet the need of one that is ill or dying. As a result, promoting self-efficacy among family caregivers of HIV/AIDS patients is a key process for achieving patient-focused, family-cultured medical care. Family members often feel stressed and overwhelmed by the experience of caregiving, and they often express feelings of powerlessness. Thus increasing their confidence in their ability to know what to do and how to provide care can go a long way in reducing their stress, since self-efficacy refers to a person's confidence in how well he/she can accomplish a task or group of tasks.

At present, few Nigerians have access to basic HIV/AIDS care, support and treatment services. It was estimated that there were 1.8 million estimated number of deaths due to AIDS and 2.6 million new infection in 2009 worldwide, 72.2% (1.3 million) and 69.2% (1.8 million) occurred in Sub-Saharan Africa. A large proportion of these cases occurred in Nigeria. With the current National prevalence of 4.1%, the number of people infected is estimated at about 3.1 million. This means that Nigeria still has the second largest number of PLWHA in Sub-Saharan and the highest in West African sub-region. Currently, about 1.5 million people including 212,720 children are still in need of AIDS treatment. Around 636,000 people were estimated to require Antiretroviral Therapy (ART) in Nigeria (WHO/UNAIDS 2005), and only 31,794 were receiving this at 71 treatment sites (WHO/UNAIDS 2005).

According to reports, 25 million people have died from AIDS in the 25 years of occurrence (UNAIDS 2006). A further 40 million men, women and children are now living with HIV. According to the global report of the AIDS epidemic by the Joint United Nations Programme on HIV/AIDS (UNAIDS 2006), promising developments have been seen in recent years in global affairs to address the AIDS epidemic, but the number of people living with HIV continues to grow, as does the number of deaths due to AIDS. Nevertheless, although the situation is improving among many groups in Nigeria and Africa, large numbers of infections occur every year and the number of deaths is increasing.

As the epidemic continued, so is the stress experienced by informal caregivers, thereby causing discrimination of those who are indirectly touched or affected by HIV/AIDS. Those particularly important to PLWHA and who are likely to be affected by this secondary epidemic are informal caregivers. These caregivers vary in the types of tasks performed, the amount of time devoted to caregiving and living arrangements (i.e. same or separate household).

The role of the informal caregiver is thus very significant for PLWHA. Informal caregivers perform a variety of roles that help people with AIDS to adhere to treatment regimens, avoid unnecessary hospital admissions, reduce the need for formal caregivers, remain at home longer and maintain a good quality of life. Traditionally, family members have served as the primary caregivers for seriously ill individuals. Because HIV care involves more diverse social networks, many HIV-positive individuals have redefined family boundaries to include lovers, friends and other “chosen kin”. Thus, a chronic illness such as HIV/AIDS affects not only the lives of those suffering from the disease, but also those of family members who care for them.

The caregiving exerts increased AIDS financial, physical and emotional responsibility on family members who care for their relatives with HIV/AIDS (Rees, O'Boyle & Mac Donagh 2011). Family members deal with extensive coordination of care, including symptom management, disability, mobility and dressing for the affected person(s). In the face of increasing challenges and responsibilities, caregivers often feel tired, isolated and overwhelmed, because they lack support, training, information and a sympathetic ear that are necessary to care for their relatives with HIV/AIDS (Rees *et al*. 2011). Furthermore, some family members who are employed report missing work, taking personal days off and quitting early to provide care (Rorer 1998). Caregiving activities include the provision of physical and emotional support to patients, staying awake at night to attend to relatives who are in the terminal stage of their illness and helping those with frequent bouts of diarrhea among other debilitations. They also take on household chores and assist with care of the children of the sick (for young parents) and offer counseling, mental support as well as help in organizing funerals, etc. (Akintola 2004). Some face the risk of infection with Tuberculosis because of frequent close contact with the sick relatives and also risk HIV infection if they do not use protective devices.

### Rationale for the study

Caregiving is considered a stress that is expected to have implications (usually negative) on the caregivers' well-being (Mohammed & Gikonyo, 2005). It is well established that caregiving places considerable emotional burden that is both profound and overwhelming on family caregivers, the nature and extent of which leave them at great psychological morbidity (Mohammed & Gikonyo, 2005). Many caregivers report moderate to severe sleep problems, chronic illness, depression and anxiety, lack of control over their everyday lives and adjusting to the disease progression as major sources of emotional burden.

In order to help people with chronic diseases such as HIV/AIDS, comprehensive treatment should include interventions for their caregivers. Strategies that can enhance emotional well-being of informal caregivers and reducing the risk of such illnesses like depression should be promoted.

### Objective

The main objectives is to examine whether social support seeking (SSS) and self-efficacy building (SEB) strategies are effective in enhancing emotional well-being of HIV/AIDS caregivers. Also, to find out which of the two treatment strategies is more effective in enhancing emotional well-being of the caregivers and finally to establish cause–effect relationship between SSS and SEB treatments.

## Method

### Design

This was an experimental study, exploring a 3 × 2 factorial design. It involved two treatment groups and control or no treatment group. The row consists of SSS treatment, SEB treatment and the control. While the column consists of gender labeled as male and female.

### Population

The population consists of informal or family caregivers of PLWHA at the President's Emergency Plan for AIDS Relief (PEPFAR) center, University College Hospital (UCH), Ibadan, Oyo state, Nigeria.

### Sample size

Sample size of the study consists of one-hundred and sixty-five (165) caregivers, 56 males and 109 females who are taking care of the HIV/AIDS patients with the diagnosis being confirmed by physicians using formula to determine the difference between two proportions, with the expected power of 90%, using a study where proportion of clients in intervention and control groups based on their responses to the instruments administered were (58%) and (32%), respectively.

### Sampling method

Purposive sampling and randomization methods were used to select participants and assigned participants in groups. The purposive sampling technique was used because selection of sample from the population of the study was done according to the purpose of the study. Random sampling was used to select final participants. This was done by making sure that all the participants were given equal chance of participation. The researcher used a table random sampling where participants were allowed to pick yes or no wrapped in papers.

## Setting

The study was carried out in 2009 at the PEPFAR center, UCH, Ibadan, the first and the largest federal teaching hospital in Nigeria.

### Data collection

Data collection for the study was carried out using three instruments which consisted of four sections. Section A consisted of several items tapping respondents’ socio-demographic variables, such as age, sex, family, socio-economic status, occupation, etc.

### Social support scale

Social support scale (SSS) was measured using a multidimensional scale of perceived social support development by Zimet, Dahlem, Zimet, and Farley (1988). It was used to measure the degree to which respondents felt satisfied with evaluable social support and sources of their support.

It is a 12-item format scale which is scored on five points ranging from 1 (strongly disagree) to 5 (strongly agree). The items on the scale were loaded into factor groups relating to the source of the social support namely family (fam), friends (fri) or significant other (so). Higher scores indicated high level of satisfaction with social support while low scores indicated low level of social support.

### The general self-efficacy (GSE) scale

This scale was developed by Scholz, Gutiérrez Doña, Sud, and Schwarzer (2002). It was designed to assess the general sense of perceived self-efficacy in general population to predict coping with daily hassles as well as adaptation after experiencing all kinds of stressful life events. It is a 10-item scale that measures goal-setting, effort investment, persistence in face of barriers and recovery from setbacks. It has a cronbach alpha range from 0.76 to 0.90. It is arranged on a four-point scale with pre-assigned value ranges of 4 (Exactly true), 3 (moderately true), 2 (hardly true) and 1 (not at all true). Higher scores indicate higher perceived self-efficacy while lower scores denote lower perceived self-efficacy.

## Emotional well-being scale

Emotional well-being was measured here using the General Health Questionnaire (GHQ). It is a 28-item scale developed by Goldberg, Gater, Sartorius, Ustun, Puccinelli, Gureje *et al*. 1997.

The scale is designed to measure individuals’ well-being in respect of their emotional disturbances. The scale has four subscales which include somatic symptoms 7 items, social depth 7 items, anxiety and insomnia 7 items and social depression 7 items. The authors reported Cronbach Coefficient alpha between 0.82 and 0.86. Jones, Rona, Hooper, and Wesseley (2006) and Idemuoha (2002) reported alpha coefficient of 0.75 in a comparative assessment of psycho-spatial characteristics of youth in Ibadan city. Gureje and Obikoya (1990) reported a sensitivity value of 67.0 and specificity of 74.0 among Nigerians in Primary Health Care (PHC) services. The scale is rated on a four-point scale with pre-assigned value ranges from better than usual (4), same as usual (3), worse than usual (2) and much more than usual (1). Higher scores indicate low level of well-being or emotional disturbances while low scores indicate good level of emotional well-being.

## Treatment

The caregivers 165 in number were purposefully selected and randomly assigned to treatment groups and control thus SSS – 55; SEB – 55 and control group – 55. The participants were exposed to treatment for a period of eight (8) weeks, while the control was met weekly without any treatment.

## Hypotheses

1. There will be no significant treatment effects in the emotional well-being of the participants in the experimental groups (SSS and SEB Treatment Strategies) and control group.
2. There will be no significant difference in the emotional well-being of the participants in the experimental group (SSS and SEB Treatment Strategies).

### Ethical consideration

Ethical clearance was obtained from the University of Ibadan/UCH ethics committee and ethical considerations were observed throughout the study.

### Data analysis

ANCOVA statistical tool was used to analyze the data collected. This was used to establish the significant treatment effects with the pretest as covariate.

## Results

The findings of this study with particular reference to the hypotheses are discussed.

The first hypothesis states that there will be no significant treatment effects in the emotional well-being of the participants in the experimental groups (SSS and SEB Treatment Strategies) and control group.

Table 1 shows the result of the pair comparison of the adjusted *Y*-means of the treatment groups (SSS-*X* = 60.08; SEBT-*X* = 55.891 and control group (*X* = 38.56). The results of the findings show that the adjusted *Y*-means scores of the two treatments were better than the control group meaning that there was effectiveness of the two treatments over the control.

**Table 1.** Pretest (*X*-means) and post-test (*Y*-means) of subjects exposed.

Treatments	Pretest mean	Pro-test mean
SSS	60.085	53.453
SEB treatment	55.897	56.105
Control	38.568	40.878

Table 2 reveals a significant treatment effect in the emotional well-being of the participants exposed to SSS strategy and SEB strategy compared to the control group; *F*(2,159) = 20.79; *P* < .05. This again indicates that the two treatments were effective in enhancing emotional well-being of the participants.

**Table 2.** Summary of ANCOVA of the experimental groups and control on emotional well-being.

Source of variation	DF	Sum of squares	Mean squares	*F*	Sig	Remark
Treatment	2	370.478	185.239	20.79	0.000	S
Groups	1	2.820	2.820	0.32	0.768	NS
Interaction	2	21.860	10.930	1.23	0.654	NS
Residual	159	1416.690	8.910			
Total	164	1811.848				

Hypothesis Two: The hypothesis states that there will be no significant difference in the emotional well-being of the participants in the experimental group (SSS and SEB Treatment Strategies).

Table 3 shows the result of the pair comparison of the adjusted *Y*-means of the treatment groups. The SSS group was superior to the SEB group in enhancing emotional well-being of the participants in the study.

**Table 3.** Pretest (*X*-means) and post-test (*Y*-means) of subject.

Treatment	Pretest mean	Post-test mean
SSS treatment	62.572	55.982
SEB treatment	57.782	59.466

The result from Table 4 reveals a significant treatment effect in the emotional well-being of the participants exposed to SSS and those in the SEB treatment; *F*(1,106) = 10.03, *P* < .05. This indicates that the two treatments were effective in enhancing emotional well-being of the participants. However, the result showed SSST mean score (*X* = 59.29) and SSBT (*X* = 58.20), indicating the superiority of SSS treatment over SEB treatment in improving the emotional well-being of informal HIV/AIDS caregivers.

**Table 4.** Summary of ANCOVA of the experimental groups on emotional well-being.

Source of variation	DF	Sum of squares	Mean squares	*F*	Sig	Remark
Treatment	1	124.17	124.17	10.03	0.00	S
Groups	1	6.02	6.02	0.49	0.649	NS
Interaction	1	17.10	17.10	1.38	0.538	NS
Residual	106	1312.28	12.38			
Total	119	1459.57				

## Discussion

This study aimed at finding out the relative efficacy of SSS and SEB in the management of emotional well-being of caregivers of people suffering from HIV/AIDS in Ibadan, Oyo state, Nigeria, with the aim of whether the independent variables can be used to enhance emotional well-being of caregivers of PLWHA and which of the strategies is more effective. The result of the findings revealed that the treatment effects were very effective and superior to the control group in enhancing emotional well-being of the participants. This finding corroborates the study of Spiegel, Butler, Gieses-Davis, Koopman, Miller, DiMiceli, *et al*. (2007), who found that social support interactions with persons suffering from chronic illness have resulted in improved adaptations. Similarly, Ostrow, Whitaker, Frasier, Cohen, Wan, Frank, *et al*. (1991) found a strong relationship between level of distress and disclosure of HIV status and availability of social support. Rees and Kaplan (2000) found that both social support and spirituality had inverse relationships with worry about health among people with HIV. The finding lend credence to the earlier study and submission of Baumeister, Slavsky, Muraven and Tice (1995), Rice (1998) and Vohs and Heatherton (2000) study which found a significant relationship between self-efficacy and emotional well-being of HIV/AIDS patients’ caregivers. These studies bear clear evidence that psychological therapies are very essential for sustenance of mankind's emotional well-being.

It was further observed that there were significant treatment effects of these two treatment groups when compared with each other and social support treatment strategy appeared as more effective than SEB strategy in enhancing emotional well-being of the participants. These findings corroborate Stajduhar and Davies (1998) in their study on palliative care at home. They found that there was a strong relationship between social support and emotional well-being of caregivers. Research carried out in Mumbai by D'Cruz (2002) and in line with the findings stated that caregivers need support to cope with the demand and outcomes of their role. Another similar study found that social support is positively related to better health outcomes, better coping and less negative effects of stress (Bert 2006).

Research on self-efficacy has focused on the individual's capacity to monitor and modify behavior, cognition and affect in order to achieve a goal (Efklides, Niemivirta & Yamauchi 2002). This finding corroborates Katz and Campbell (1994), and Williams, Grow, Freedman, Ryan and Deci (1996) which found a significant relationship between self-efficacy and emotional well-being of HIV/AIDS caregivers. The probable reason why this is so may be that social support has an all-encompassing ability to stabilize the caregivers’ emotional state more than self-efficacy.

### Implications of the study

The study has found that social support and self-efficacy strategies were very effective in enhancing emotional well-being of the participants in the study. It is believed that these two techniques will serve as key processes for achieving patient-focused, family-centered care as well as patients' outcomes care based on the social support and SEB training that had been given to the participants.

### Recommendations

Based on the findings of this study, it was recommended that strong link should be created between the different organizations in care and support of PLWHAS in communities and taken care of. Counseling should be extended to informal caregivers especially with regard to their emotional and psychological needs. Finally labor organizations and parastatals should implement work place support groups as a way to mitigate the impact of informal caregiving on workers. Thus, counseling, linkages and information services can be employed and sponsored in work place for informal caregiver support groups.

## Conclusion

The results reveal the appropriate psychological techniques that can be used by Nigerian clinical psychologists and other members of the health care team as an adjunct to the management of emotional well-being of the caregivers. In addition, the research has empirically demonstrated how the therapeutic techniques could be effectively used in the management of caregivers' emotional well-being. The mastery of the techniques by the caregivers/participants who participated in this study enabled them to be in control of their peculiar circumstances.
